# Supplementary material for: Exosome-mediated stable epigenetic repression of HIV-1
Source: Nat Commun. 2021 Sep 20;12:5541. doi: 10.1038/s41467-021-25839-2 (PMC8452652; doi:10.1038/s41467-021-25839-2)
Supplement: Supplementary file 1 — Supplementary Information [file 41467_2021_25839_MOESM1_ESM.pdf]

Supplementary Figures

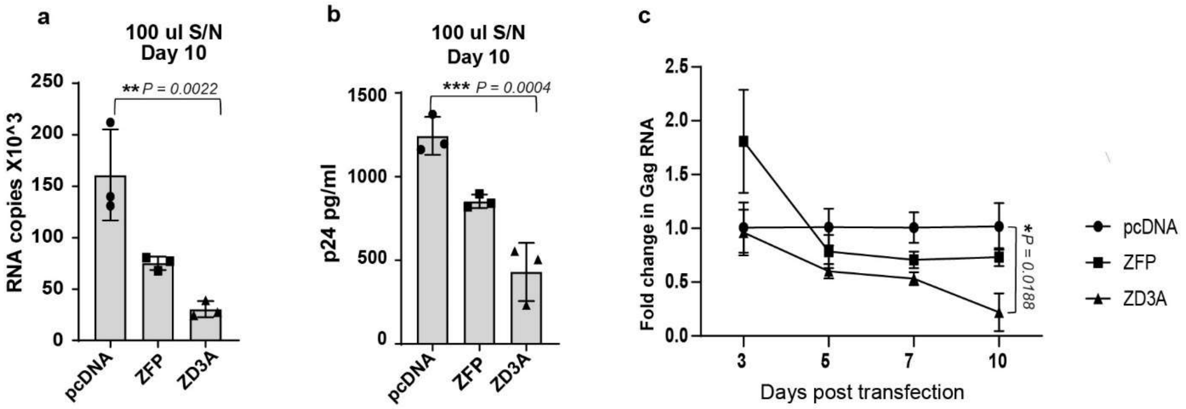

Supplementary Figure 1. Confirmation of ZD3A driven repression. After10 days of transfection with control, ZFP or ZD3A plasmids, culture supernatant was collected, and **a** copy number of viral RNA was measured. **b** Viral protein p24 was measured through ELISA. **c** Gag RNA was measured from transfected cells over the course of 10 days at indicated timepoints. In **a-c** data represents mean ±SD of triplicate treated samples. For **a** and **b** P value was determined by one-way ANOVA followed by Tukey's post hoc test for multiple comparison. For **c,** P value was determined by two-way ANOVA followed by Tukey's post hoc test for multiple comparison. Source data are provided in source data file.

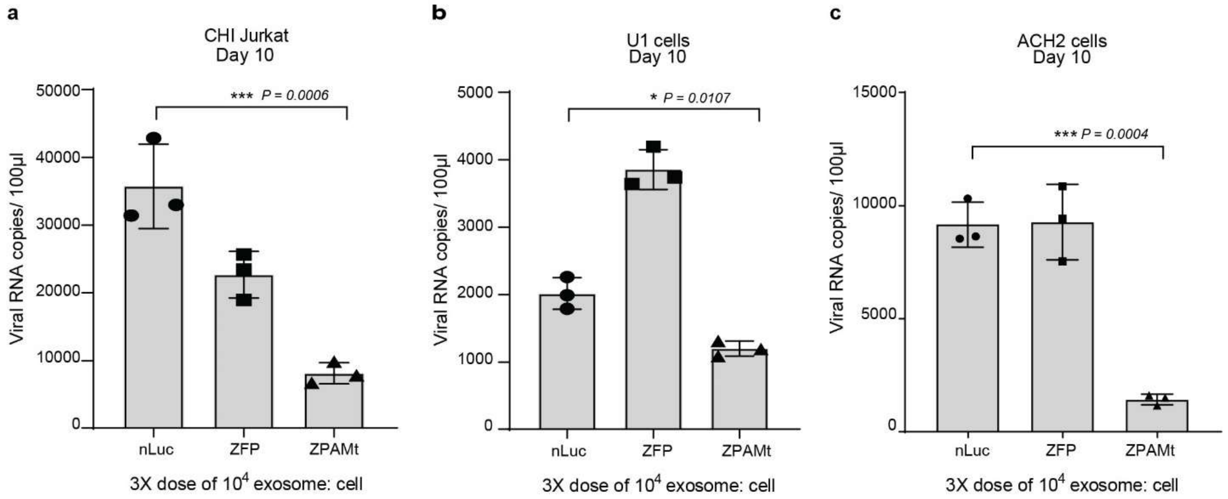

Supplementary Fig 2. Exosomal ZPAMt lowers viral production from different cell lines. Viral copy number was assayed via RT-qPCR from supernatant of exosome treated **a** CHI Ju cells **b** TNFα activated U1 cells and **c** TNFα activated ACH2 cells. Data represents mean ± of samples treated in triplicate, P values were calculated using ordinary one-way ANOVA followed by Tukey's post-hoc test for multiple comparison. Source data are provided in source data file.

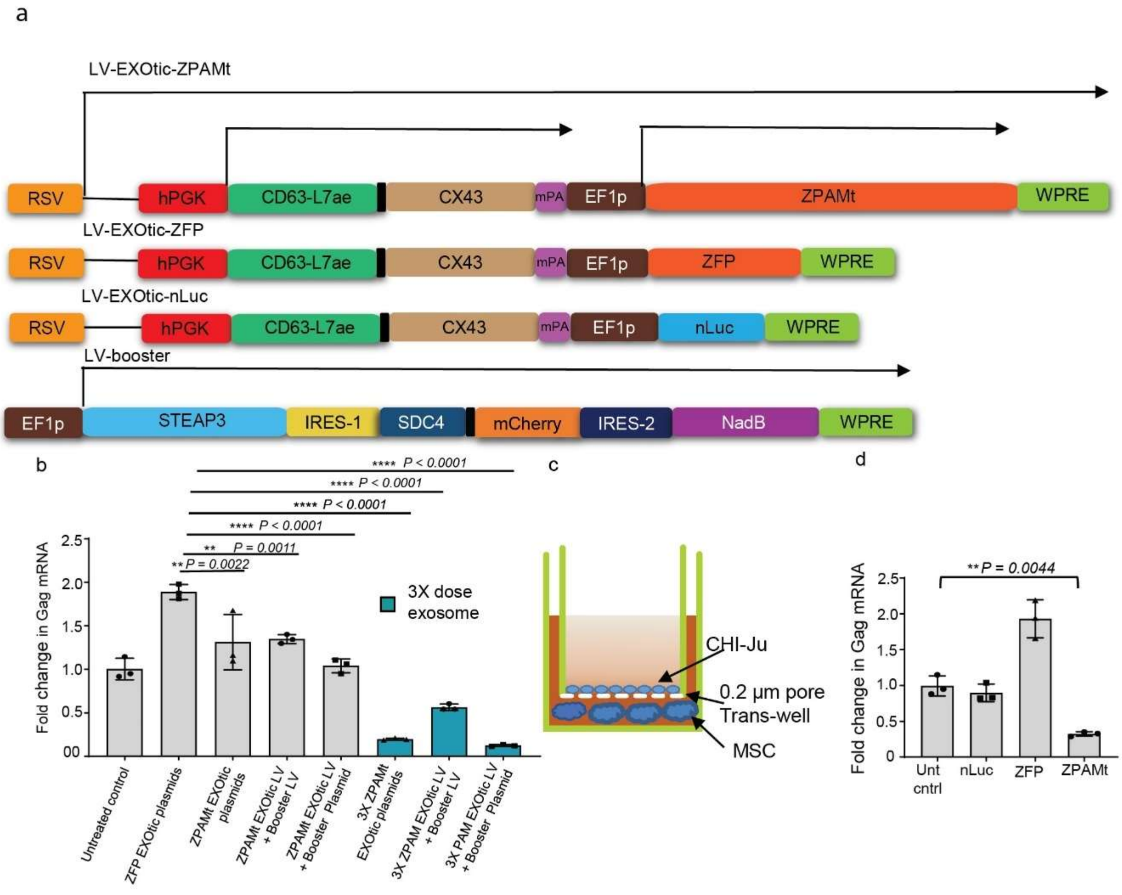

Supplementary Figure 3. Adoption of EXOtic device plasmids for generation of stable exosome producer cells. a Schematics depicting design of LV plasmids for producer and booster LV. First three plasmids show expression of complete genome under RSV promoter terminating after WPRE for packaging in lentivirus vectors. Cells were transduced with either of these LVs produced CD63-L7Ae and Connexin43 S368A (CX43) proteins which facilitates exosome packaging and release of EF1p expressed mRNAs containing the C/D_{box} in the 3'UTR. The fourth plasmid represents adoption of EF1α promoter driven tri-ORF exosome booster plasmid to be packaged in a LV. **b** Exosomes were isolated from HEK cells transduced with combination of Lentiviral vectors in **a** or transfected with EXOtic component plasmids. Exosomes were added in ratio of 10000 per cell to CHI-Ju cells and RT-qPCR for Gag was done at after 10 days of exosome treatment **c**. Cartoon depiction of a trans-well assay with MSC seeded at the bottom and transduced with the nLuc, ZFP or ZPAMt and booster LV described in **a.** The top well contained the CHI-Ju **c.** Effect of 10 days of co-culture of exosome producing MSC with cargo denoted in graph on CHI-Ju cells in the form of Gag RNA expression. For **b** and **d** data presents mean ± SD of samples treated in triplicate, significance was calculated using ordinary one-way ANOVA followed by

Tukey's post-hoc test for multiple comparison. ****$p \leq 0.0001$. Source data are provided in source data file.

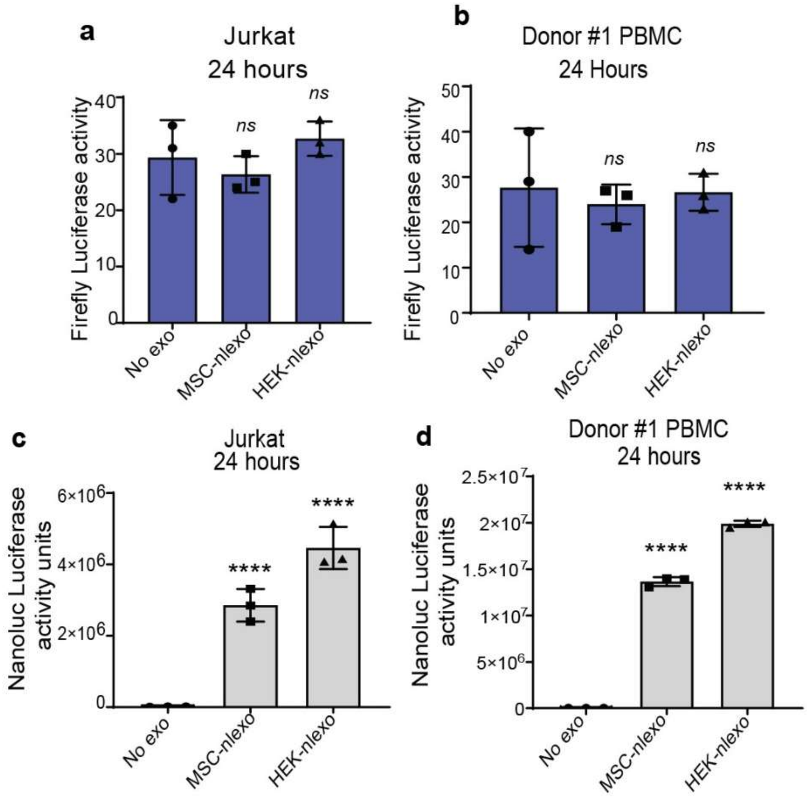

Supplementary Figure 4. Active packaging of nLuc in exosomes from LV-EXOtic-nLuc transduced, exosome producer cells. hTERT-MSC having stably integrated FLuc gene and HEK293T cells were transduced with LV_EXOtic-nLuc. Exosomes derived from these cells i.e., MSC-nLuc-exo and HEK-nLuc-exo were exposed to **a** Jurkat cells and **b** Donor#1 derived PBMC and Fluc activity was measured and compared to unexposed cells (no exo) **c** and **d** nluc activity was also measured from the same set of cells and compared to unexposed cells (no exo), revealing insignificant FLuc activity and significant nLuc activity. Data presents mean ± SD of samples treated in triplicate; significance was calculated using ordinary one-way ANOVA followed by Tukey's post-hoc test for multiple comparison. ****$p \leq 0.0001$ Source data are provided in source data file.

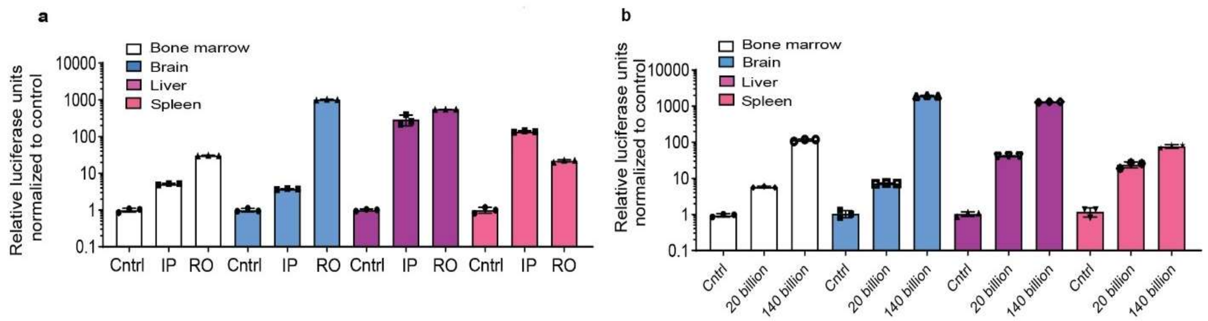

Supplementary Figure 5. *In vivo* distribution of reporter exosome cargo. a nLuc activity measured from indicated organs in euthanized NSG treated mice 4 hours after 20 x 10^9 nLuc exosome administration via IP or RO route. Untreated HEK293T derived exosome injected mice served as control. **b** Concentration dependent change in nano-luciferase (nLuc) activity measured from lysates of indicated organs in euthanized NSG mice 4 hours of after RO administration of nLuc packed exosomes. PBS injected mouse was used as a control. The lysates from each mouse organ were analysed for luciferase activity in triplicates, where 1 mouse was used per condition. . Data represents means ± error bars indicate standard deviation of triplicate samples. Source data are provided in source data file.

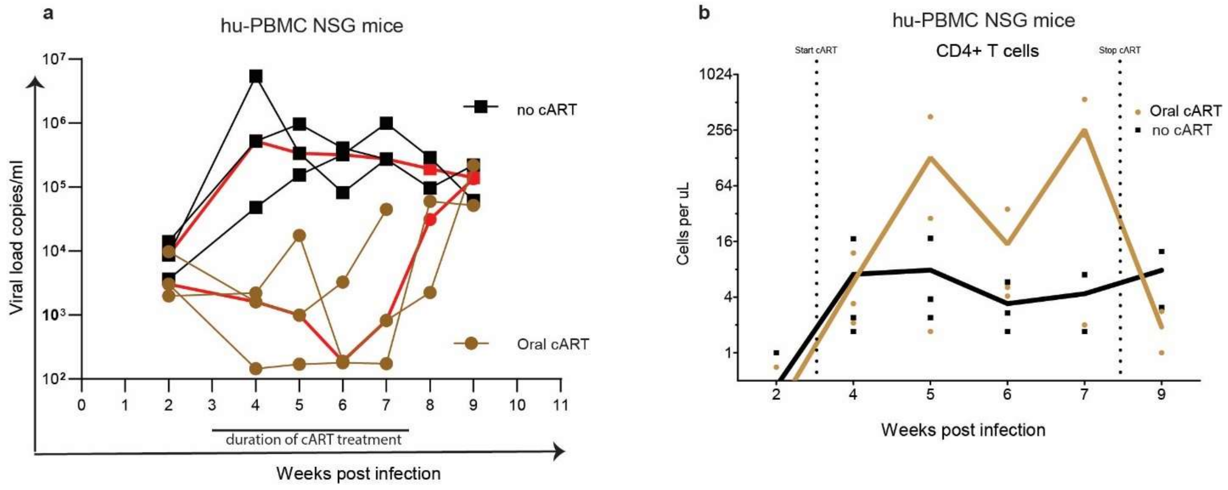

Supplementary Figure 6. Preliminary studies on oral cART efficacy. HIV-1 infected hu-PBMC NSG mice subjected to 4 weeks of oral ART treatment or left untreated. **a** Viremia levels were measured periodically from oral cART treated mice and untreated mice. Red line indicates median viremia level. **b** Peripheral human CD4+ T cells were also counted using flow cytometry from these mice periodically. Trendline in **b** indicates mean of absolute counts of CD4+ in mice of each category**.** Three mice were used in each category. Source data are provided in source data file.

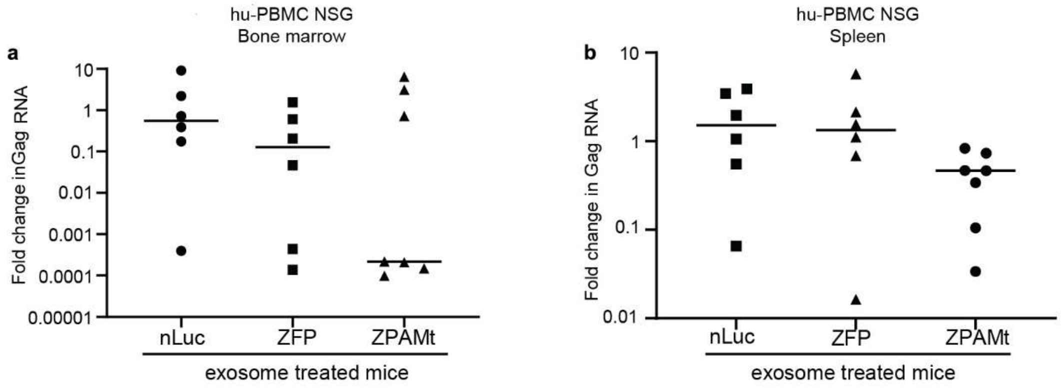

Supplementary Figure 7. Organ specific changes in viral load in hu-PBMC-NSG mice. RT-qPCR for Gag RNA expression in nLuc/ZFP/ZPAMt exosome-treated mice from viral reservoir within the (**a**) bone marrow and (**b**) spleen after 10 weeks of HIV infection. Source data are provided in source data file.

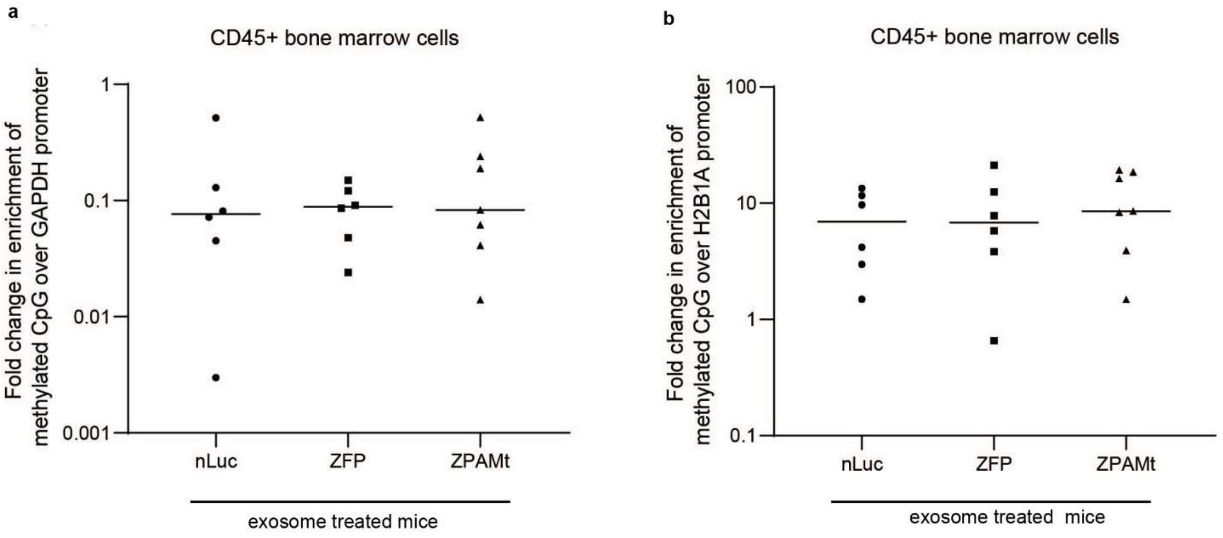

Supplementary Figure 8. Specific methylation of HIV LTR via ZPAMt exosomes. Bone marrow derived CD45+ cells from HIV infected hu-PBMC-NSG administered with either nluc, ZFP or ZPAMt exosomes were checked for enhanced CpG methylation on **a** GAPDH promoter **b** H2B1A promoter. Source data are provided in source data file.

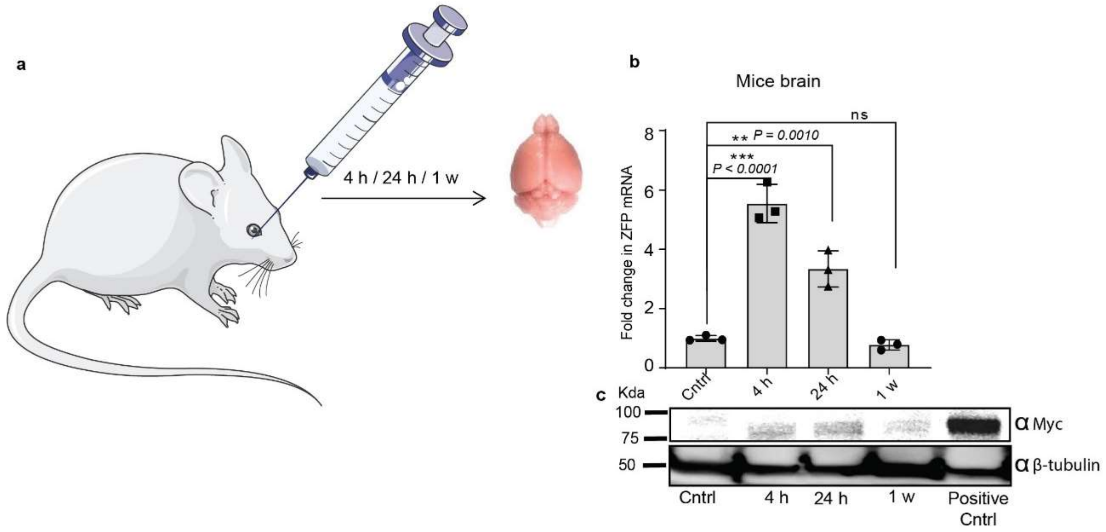

Supplementary Figure 9. Exosome mediated delivery of ZPAMt in brain of NSG mice. **a**. Schematic for methodology of testing delivery and expression of ZPAMt, wherein 100 x 10^9 ZPAMt packed exosomes were RO injected in NSG mice and 1 mouse was euthanized at 4 h, 24 h, or 1 week time points. Untreated mouse was used as control. **b** RNA from whole brain lysate from all mice euthanized at different time-points was extracted and RT-qPCR for ZFP mRNA was performed to check delivery of ZPAMt-C/D_{box} via exosomes. The error bars indicate standard deviation of triplicate treated samples. Lysate from brain of each mice was processed in triplicate. Data presents mean ± SD. P value was calculated using one-way ANOVA followed by Tukey's post hoc test for multiple comparison test. **c** Western blot for myc tagged ZPAMt protein from whole brain lysate from all mice euthanized at different point. Lysate from ZPAMt transfected HEK293T were used as positive control. β-tubulin was used as loading control. Representative blot of n = 2 technical replicates from one mouse per condition. Source data are provided in source data file.

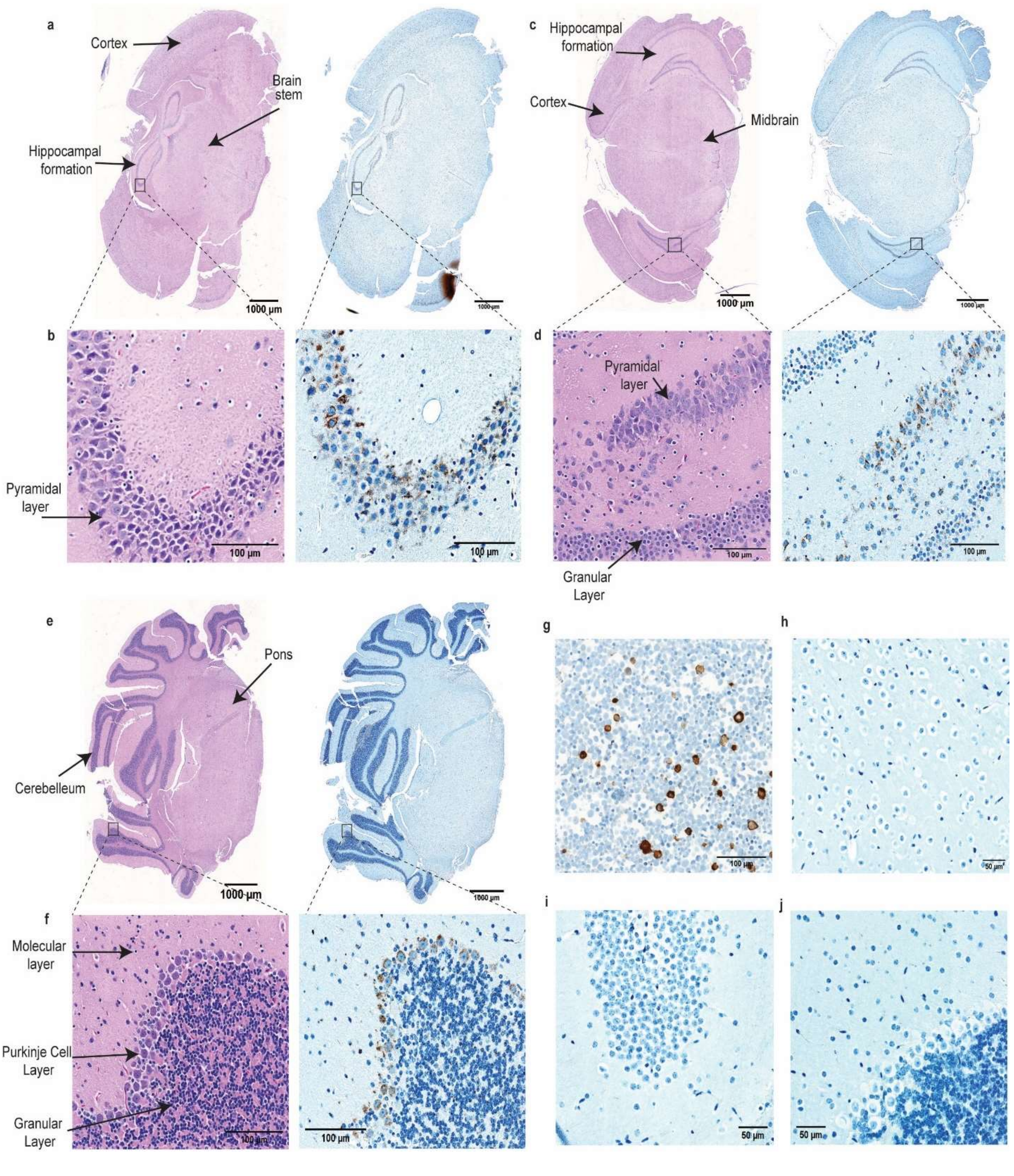

Supplementary Figure 10. **p24 Immunohistochemistry (IHC) staining in coronal brain sections indicates p24+ cells in all regions of the brain assayed**. Level I coronal sectioning **a** and **b** with hematoxylin and eosin (H&E) staining (left) and p24-DAB and hematoxylin staining (right). **a** Full brain section with **b** its corresponding region of magnification. Level I section predominantly consisted of the cortex and the brain stem. Level II coronal sectioning (**c** and **d**). **c**

Full brain section with **d** its corresponding region of magnification. Level II sections predominantly consisted of the cortex and the midbrain. Level III coronal sectioning (**e** and **f**). **e** Full brain section with **f** its corresponding region of magnification. Level III sections predominantly consisted of the cerebellum and the Pons. **g** Positive control for the p24 antibody using the chronically infected Jurkat cell line in a paraffin block and (**h-j**) are magnified regions of a non-humanized uninfected NSG mouse from a **h** level I, **i** level II and **j** level III section indicating no non-specific or background staining with the p24 primary antibody. Images **a-f** are from one huCD34+-NSG brain section and is representative of 17 individual huCD34+-NSG mice brain sections from one independent experiment. Image **g** is representative of one individual cell block section from the chronically infected Jurkat cell line. Images **h-j** are representative of one NSG mice from one independent experiment. Source data are provided in source data file.

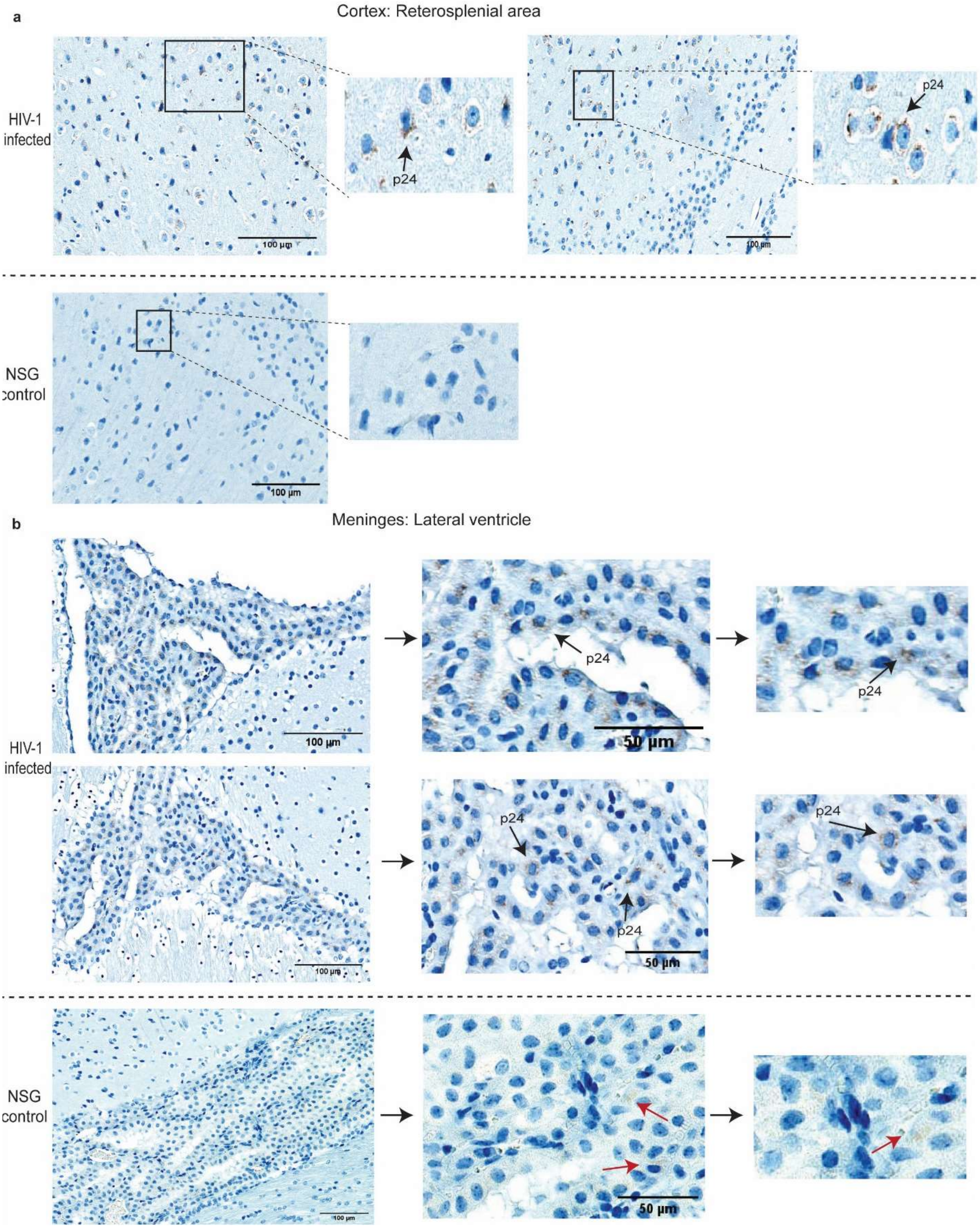
a
Cortex: Reterosplenial area
HIV-1 infected
p24
p24
100 µm
100 µm
NSG control
100 µm
b
Meninges: Lateral ventricle
HIV-1 infected
p24
p24
100 µm
50 µm
p24
p24
p24
100 µm
50 µm
NSG control
100 µm
50 µm

Supplementary Figure 11 IHC staining in coronal brain sections indicates p24+ cells the cortex and in the meninges. Level I coronal sectioning (**a** and **b**) with p24-DAB and hematoxylin staining. (**a**) Level I coronal section showing the reterosplenial region within the cerebral cortex and a magnified region indicates p24+ cells (right) in HIV-1 infected mice but not in the uninfected NSG control mice. (**b**) Level I coronal section showing the lateral ventricle containing the meninges (left) and progressively magnified regions indicates p24+ cells in the meninges (left) in HIV-1 infected and in an uninfected NSG control mice. Black arrows denote p24+ staining and red arrows indicate diffuse background staining in the uninfected NSG control. Images in **a** are from two individual huCD34+-NSG brain sections and are representative of 17 individual huCD34+-NSG mice brain sections from one independent experiment, where the reterosplenial region was observable. The NSG control image is representative of one NSG mouse, from one independent experiment. Images in **b** are from two individual huCD34+-NSG brain sections and are representative of three individual huCD34+-NSG mice brain sections from one independent experiment, where the lateral ventricle was observable. The NSG control image is representative of one NSG mouse, from one independent experiment.

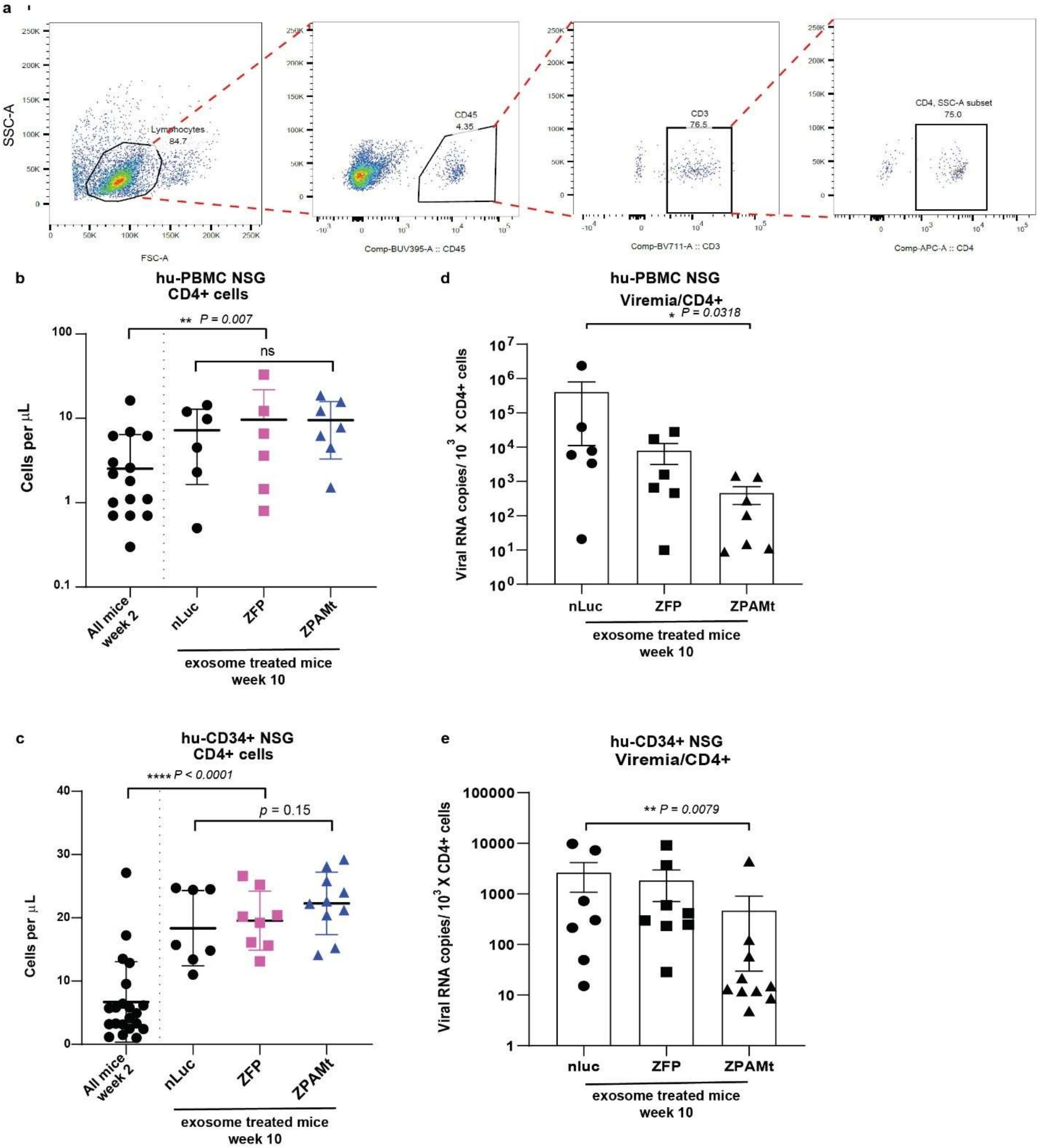

Supplementary Figure S12. CD4+ gating strategy, absolute count and baselining a Flow cytometry gating strategy for the detection of CD4+ T cells. Lymphocytes were gated for BUV395 conjugated anti-CD45 stained cells. From CD45+ cells, positively stained cells for BV711 conjugated anti-CD3 stained cells were gated. Finally, APC conjugated anti-CD4 stained cells were detected from CD3+ cells. Absolute CD4+ cell count from all the mice at 2 weeks post infection and at week 10 from exosome packaged nanoluc/ZFP or ZPAMt treated **b** hu-PBMC NSG mice and **c** hu-CD34+ NSG mice are plotted. For **b** and **c** the line and error bars indicate mean ±standard deviation. Viremia at week 10 as depicted in Fig. **4b** and Fig. **5b** was baselined with CD4+ cell count at week 10 and plotted for **d** hu-PBMC NSG mice and **e** hu-CD34+ NSG

mice. For **d** and **e** data resents mean ± standard error of the mean, P values were calculated using Kruskal-Wallis test. ****$P \leq 0.0001$. Source data are provided in source data file.
