# Supplementary material for: Exosome-mediated stable epigenetic repression of HIV-1
Source: Nat Commun. 2021 Sep 20;12:5541. doi: 10.1038/s41467-021-25839-2 (PMC8452652; doi:10.1038/s41467-021-25839-2)
Supplement: Supplementary file 3 — Description of Additional Supplementary Files [file 41467_2021_25839_MOESM3_ESM.pdf]

Description of Additional Supplementary files

File name: Supplementary Data 1

Description: Sequence of gBlocks, and oligomers used in this study. Supplementary data 1 provides sequences of all the gBlocks used for cloning fusion constructs and C/D_{box} RNA domain as DNA. Supplementary table 1 also provides sequences of primers used for Polymerase Chain Reaction (PCR) as described in methods section.
