# Supplementary material for: Exosome-mediated stable epigenetic repression of HIV-1
Source: Nat Commun. 2021 Sep 20;12:5541. doi: 10.1038/s41467-021-25839-2 (PMC8452652; doi:10.1038/s41467-021-25839-2)
Supplement: Supplementary file 5 — Reporting Summary [file 41467_2021_25839_MOESM5_ESM.pdf]

nature research

Corresponding author(s): Kevin Morris
Last updated by author(s): Jul 15, 2021

Reporting Summary

Nature Research wishes to improve the reproducibility of the work that we publish. This form provides structure for consistency and transparency in reporting. For further information on Nature Research policies, see our Editorial Policies and the Editorial Policy Checklist.

Statistics

For all statistical analyses, confirm that the following items are present in the figure legend, table legend, main text, or Methods section.

n/a	Confirmed	
☐	☒	The exact sample size (n) for each experimental group/condition, given as a discrete number and unit of measurement
☐	☒	A statement on whether measurements were taken from distinct samples or whether the same sample was measured repeatedly
☐	☒	The statistical test(s) used AND whether they are one- or two-sided<br>*Only common tests should be described solely by name; describe more complex techniques in the Methods section.*
☒	☐	A description of all covariates tested
☒	☐	A description of any assumptions or corrections, such as tests of normality and adjustment for multiple comparisons
☐	☒	A full description of the statistical parameters including central tendency (e.g. means) or other basic estimates (e.g. regression coefficient) AND variation (e.g. standard deviation) or associated estimates of uncertainty (e.g. confidence intervals)
☐	☒	For null hypothesis testing, the test statistic (e.g. F, t, r) with confidence intervals, effect sizes, degrees of freedom and P value noted<br>*Give P values as exact values whenever suitable.*
☒	☐	For Bayesian analysis, information on the choice of priors and Markov chain Monte Carlo settings
☒	☐	For hierarchical and complex designs, identification of the appropriate level for tests and full reporting of outcomes
☒	☐	Estimates of effect sizes (e.g. Cohen's d, Pearson's r), indicating how they were calculated

Our web collection on statistics for biologists contains articles on many of the points above.

Software and code

Policy information about availability of computer code

Data collection	Following software were used to record data<br>1.Gatan Microscopy Suite Software (GMS 3) for TEM<br>2. BD FACs Diva 8.0.1 Software for Flow Cytometry acquisition<br>3. NTA 3.1 software for Nanosight Tracking analysis
Data analysis	Following software/tools were used to analyze data<br>1. Roche Lightcycler 96 V1.1 and BioradCFX manager V2.1 for RT-qPCR<br>2. Imagelab software for PAGE and agarose gel imaging V5.2.1<br>3. Promega Glomax explorer v3.2.3 for luciferase activity readouts.<br>4. SoftMax Pro 7 Software for spectroscopy based readouts.<br>5. QuPath version 0.2.24 to analyse IHC slides<br>6. GraphPad Prism v8.3.0 for plotting graphs.

For manuscripts utilizing custom algorithms or software that are central to the research but not yet described in published literature, software must be made available to editors and reviewers. We strongly encourage code deposition in a community repository (e.g. GitHub). See the Nature Research guidelines for submitting code & software for further information.

Data

Policy information about availability of data

All manuscripts must include a data availability statement. This statement should provide the following information, where applicable:

- Accession codes, unique identifiers, or web links for publicly available datasets
- A list of figures that have associated raw data
- A description of any restrictions on data availability

The source data underlying Figs 1 - 5 and supplementary Figs 1 -9 and 12 are provided in source data file. All other relevant data supporting key finding of this study are available within the article and its supplementary files or from corresponding authors upon reasonable request.

Field-specific reporting

Please select the one below that is the best fit for your research. If you are not sure, read the appropriate sections before making your selection.

[x] Life sciences [ ] Behavioural & social sciences [ ] Ecological, evolutionary & environmental sciences

For a reference copy of the document with all sections, see nature.com/documents/nr-reporting-summary-flat.pdf

Life sciences study design

All studies must disclose on these points even when the disclosure is negative.

Sample size	Since this was the first study to determine the effect of exosome-packaged ZPAMt in mice models of HIV infection, we used the effect size from our in-vitro experiments. As per fig 2D in-vitro data showed a large effect size of 65% viral suppression. A total sample size of 24 mice was estimated given alpha error probability of 0.05, 80% study power, effect size of 0.55, for 3 groups and 5 repeated measurements using G*Power software v3.1 (Faul, F. et al. (2009). Behavior Research Methods, 41, 1149-1160). The sample size (n) can be found in the figure legends.<br>Previously published paper examining the effects of exosome delivery cargo also used similar sample size. (Kojima, R. et al. Nat Commun 9, 1305, (2018)).
Data exclusions	The following were conditions of exclusion were pre-established. Mice under one or both were not included in experimentation and data shown in Fig. 4-5 and supplementary figs. 6 to 12.<br>1. Undetectable HIV-1 levels in blood at week 2 time point.<br>2. Undetectable en-grafted human cells at week 2 time point.
Replication	As described in figure legends, experiments were repeated three times to ensure reproducibility.
Randomization	Samples and mice were all randomly allocated to different experimental groups in this study. No specific randomization protocol has been used.
Blinding	Not applicable as the extracted results are objective in this study

Reporting for specific materials, systems and methods

We require information from authors about some types of materials, experimental systems and methods used in many studies. Here, indicate whether each material, system or method listed is relevant to your study. If you are not sure if a list item applies to your research, read the appropriate section before selecting a response.

Materials & experimental systems

n/a	Involved in the study	
[ ]	[x]	Antibodies
[ ]	[x]	Eukaryotic cell lines
[x]	[ ]	Palaeontology and archaeology
[ ]	[x]	Animals and other organisms
[ ]	[x]	Human research participants
[x]	[ ]	Clinical data
[x]	[ ]	Dual use research of concern

Methods

n/a	Involved in the study	
[x]	[ ]	ChIP-seq
[ ]	[x]	Flow cytometry
[x]	[ ]	MRI-based neuroimaging

Antibodies

Antibodies used	Following are the antibodies we have used<br>1. anti-Myc antibody (2276) (Cell Signaling. catalogue no. 9B11)

2. anti-Alix mouse (3a9) monoclonal antibody (Cell Signaling, catalogue no. ab117600)
3. anti-CD63 (MX-49.129.5) mouse monoclonal antibody (Santacruz Biotechnology, catalogue no. SC5275)
4. anti-GAPDH (0411) mouse monoclonal antibody (Santacruz Biotechnology, catalogue no. SC47724)
5. anti-Tsg101(C-2) mouse monoclonal antibody (Santacruz Biotechnology, catalogue no. SC7964)
6. anti-tubulin mouse monoclonal antibody (catalogue no. abcam, ab6046)
7. Secondary Goat Anti-Mouse IgG (H + L)-HRP Conjugate (Biorad catalogue no. #1706516)
8. anti 5-methylcytosine (5-mC) (33D3) (Abcam catalogue no. ab10805)
9. BUV395-conjugated anti-human CD45 antibody Clone HI30 (BD Biosciences, BDB563792)
10. BV711-conjugated anti-human CD3 antibody Clone UCHT1 (BD Biosciences, BDB563725)
11. APC-conjugated anti-human CD4 antibody Clone RPA-T4 (BD Biosciences, BDB555349)
12. Secondary Goat Anti-rabbit IgG (H + L)-HRP Conjugate (Biorad catalogue no. #1706515)

Validation

Following are the references or webpage listing the references corresponding to antibodies listed in last tab
1. https://www.cellsignal.com/products/primary-antibodies/myc-tag-9b11-mouse-mab/2276
2. https://www.cellsignal.com/products/primary-antibodies/alix-3a9-mouse-mab/2171
3. https://www.scbt.com/p/cd63-antibody-mx-49-129-5;jsessionid=idK6lzIUjoO0-zv59V4yWTkBzOhIaIxYIUGq84O9KV5kTGxJvXUX!-1887308896
4. https://www.scbt.com/p/gapdh-antibody-0411
5. https://www.scbt.com/p/tsg-101-antibody-c-2
6. https://www.abcam.com/beta-tubulin-antibody-loading-control-ab6046.html
7. https://www.labome.com/product/Bio-Rad/170-6516.html
8.https://www.abcam.com/5-methylcytosine-5-mc-antibody-33d3-ab10805.html
9. https://www.bdbiosciences.com/us/applications/research/stem-cell-research/cancer-research/human/buv395-mouse-anti-human-cd45-hi30/p/563792
10. https://www.bdbiosciences.com/us/applications/research/t-cell-immunology/th-1-cells/surface-markers/human/bv711-mouse-anti-human-cd3-ucht1-also-known-as-ucht-1-ucht-1/p/563725
12.https://www.labome.com/product/Bio-Rad/170-6515.html

Eukaryotic cell lines

Policy information about cell lines

Cell line source(s)

HEK293T cells (available from ATCC CRL-3216™) were purchased from Life technologies now Thermo Fischer Scientific. Immortalized MSCs expressing human Telomerase Reverse Transcriptase with basal levels of GFP and Firefly luciferase were a kind gift from Dr. Carlotta Glackin at Beckman Research Institute, City of Hope National Medical Center (Nierste, B. A. Am J Blood Res 4, 73-85 (2014). Briefly, BM-MSCs were isolated from 15-week human fetal bone tissue and bone marrow was purified based on the expression of STRO-1bright/CD106+ or STRO-1bright/CD146+ expression. This STRO-1bright/CD106+ or STRO-1bright/CD146+ cell population was then immortalized with hTERT in a pBABE retroviral insertion vector and stable clones were selected with puromycin, resulting in the creation of the hTERT-MSC cell line. Jurkat (E6-1) Cells were obtained from NIH HIV reagent program (APR-177). Cell lines ACH2 and U1 were also obtained from NIH HIV reagent program with catalogue number ARP-349 and ARP-165 respectively.

Authentication

None of the cell lines were authenticated

Mycoplasma contamination

Cell lines tested negative for mycoplasma contamination.

Commonly misidentified lines
(See ICLAC register)

No commonly misidentified cell line were used in this study.

Animals and other organisms

Policy information about studies involving animals; ARRIVE guidelines recommended for reporting animal research

Laboratory animals

NOD.Cg-Prkdcscid Il2rgtm1Wjl/SzJ (NSG) mice (JAX stock #005557, Jackson Laboratory) were age- and sex- matched. NSG mice were 6 to 8 week old when engrafted with human PBMC to create hu-PBMC+NSG mice. Ten female and nine male mice were used in this cohort. For creating hu-CD34+ NSG mice, 72 hour old pups were irradiated and injected with human CD34+ haematopoietic stem cells. Fourteen female and 11 males were used in this cohort.

Wild animals

Study did not involve wild animals

Field-collected samples

Study did not involve samples collected from field.

Ethics oversight

All animal care and procedures have been performed according to protocols reviewed and approved by the City of Hope Institutional Animal Care and Use Committee (IACUC) held by the principal investigator for this application (John Burnett and Kevin Morris, IACUC 16095).

Note that full information on the approval of the study protocol must also be provided in the manuscript.

Human research participants

Policy information about studies involving human research participants

Population characteristics	Anonymous, healthy, adult donors with no identifiers for age, race, ethnicity, or gender who volunteered to donate blood at the City of Hope Apheresis Center (Duarte, CA, USA)
Recruitment	Not applicable as discarded peripheral blood samples were used.
Ethics oversight	Blood was collected under City of Hope (COH) Institutional Review Board (IRB) ref #19582. COH IRB evaluated and determined that this project does not meet the definition of human subjects research as set forth at (45 CFR 46.102 (d)(f)).

Note that full information on the approval of the study protocol must also be provided in the manuscript.

Flow Cytometry

Plots

Confirm that:

- [x] The axis labels state the marker and fluorochrome used (e.g. CD4-FITC).
- [x] The axis scales are clearly visible. Include numbers along axes only for bottom left plot of group (a 'group' is an analysis of identical markers).
- [x] All plots are contour plots with outliers or pseudocolor plots.
- [x] A numerical value for number of cells or percentage (with statistics) is provided.

Methodology

Sample preparation	Protocol followed:<br>1. Under general anesthesia,100-200l of blood was collected via Retro Orbital bleeding from mice using BSL 2+ safety practices.<br>2. Blood tubes were mixed thoroughly and 50 uL of blood into added to FACS Tubes.<br>3. Following antibody cocktail was prepared using brilliant buffer (BD Biosciences) per sample and cell marker antibodies<br>a. 50 ul of brilliant buffer per sample.<br>b. 2 ul of CD45, CD3, CD8 antibody + 5 ul CD4 antibody per sample<br>4. Antibody cocktail was added to each sample and vortexed lightly following incubation at 4C for 30 minutes.<br>5. 450 ul of BD Lyse/Fix buffer (BD Biosciences) per tube was added and vortexed lightly.<br>8. Counting beads equal to blood volume of 50 ul were added to tube and vortexed.<br>9. Tubes were incubated at room temp for 15 minutes, away from light before FACs analysis or stored in 4 deg C and dark until ready for analysis.
Instrument	BD Fortessa with 5 Laser (355nm, 405nm, 488nm, 561nm & 640nm)
Software	FLOWJO v10.7
Cell population abundance	Abundance of subpopulations varies from sample to sample therefore Counting beads (BD biosciences) to determine absolute count of each cell type.
Gating strategy	Lymphocytes were gated for BUV395 conjugated anti-CD45 stained cells. From CD45+ cells, positively stained cells for BV711 conjugated anti-CD3 stained cells were gated. Finally, APC conjugated anti-CD4 stained cells were detected from CD3+ cells

- [x] Tick this box to confirm that a figure exemplifying the gating strategy is provided in the Supplementary Information.
